# Acoustofluidic Stimulation of Functional Immune Cells in a Microreactor

**DOI:** 10.1002/advs.202105809

**Published:** 2022-03-25

**Authors:** Seunggyu Kim, Hyeono Nam, Beomseok Cha, Jinsoo Park, Hyung Jin Sung, Jessie S. Jeon

**Affiliations:** ^1^ Department of Mechanical Engineering Korea Advanced Institute of Science and Technology Daejeon 34141 Republic of Korea; ^2^ School of Mechanical Engineering Chonnam National University Gwangju 61186 Republic of Korea

**Keywords:** dynamic cell culture, immune cell response, microreactors, surface acoustic waves

## Abstract

The cytotoxic response of natural killer (NK) cells in a microreactor to surface acoustic waves (SAWs) is investigated, where the SAWs produce an acoustic streaming flow. The Rayleigh‐type SAWs form by an interdigital transducer propagated along the surface of a piezoelectric substrate in order to allow the dynamic stimulation of functional immune cells in a noncontact and rotor‐free manner. The developed acoustofluidic microreactor enables a dynamic cell culture to be set up in a miniaturized system while maintaining the performance of agitating media. The present SAW system creates acoustic streaming flow in the cylindrical microreactor and applies flow‐induced shear stress to the cells. The suspended NK cells are found to not be damaged by the SAW operation of the adjusted experimental setup. Suspended NK cell aggregates subjected to an SAW treatment show increased intracellular Ca^2+^ concentrations. Simultaneously treating the NK cells with SAWs and protein kinase C activator enhances the lysosomal protein expressions of the cells and the cell‐mediated cytotoxicity against target tumor cells. These have important implications by showing that acoustically actuated system allows dynamic cell culture without cell damages and further alters cytotoxicity‐related cellular activities.

## Graphical abstract

A microreactor system is presented, which employs surface acoustic waves to agitate fluid in a contact‐free manner. The acoustically actuated microreactor enables dynamic cell culture without cell damage by applying fluid shear stress. Reported findings reveal functional cellular changes such as elevated intracellular Ca^2+^ influx and enhanced cell‐mediated cytotoxicity in the copresence of the protein kinase C activator.

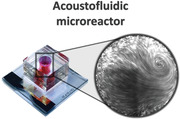

## 1 Introduction

Cells feel mechanical forces from their surrounding microenvironment, with these forces subsequently translated into intracellular signaling pathways, resulting in the regulation of various cellular functions.^[^ ^1^ ^]^ The external mechanical stimuli are transmitted to the cell nucleus by affecting membrane deformation and cytoskeleton reconstruction, and are known to have omnidirectional effects on cell differentiation, metabolism, motility and viability.^[^ ^2^ ^]^ Such mechanotransductive signaling pathways also play an important role in immune responses.^[^ ^3^ ^]^ For instance, the mechanical force exerted on T cell receptors during ligand recognition promotes the formation of immunological synapses in T cells,^[^ ^4^ ^]^ and the effector functions of natural killer (NK) cells are also tuned by cytoskeletal forces.^[^ ^5^ ^]^ In addition, extracellular forces affect mechanosensitive ion channels such as Piezo1 at the cell membrane, resulting in Ca^2+^‐dependent T cell activation.^[^ ^6^ ^]^

To meet clinical needs regarding ex vivo cultivation of cytotoxic immune cells for adoptive cancer immunotherapy,^[^ ^7^ ^]^ there has been a growing need to develop advanced cell culture systems for expanding more functional immune cells in vitro. Although numerous engineering methods including biomaterials and devices have paved the ways to regulate cellular immune functions,^[^ ^8^ ^]^ most approaches have still focused on the relation between biochemical cues (e.g., repertoire of cytokines and feeder cells) and subsequent functional alterations.^[^ ^9^ ^]^ In dynamic cell cultures, shear stress (SS) from agitated media is applied to the cells, and thus such cultures are used to harvest cytotoxic immune cells with improved anticancer potential and increased numbers.^[^ ^10^, ^11^ ^]^ As the physiological physical microenvironment alters cellular phenotypes and genotypes, mechanical stimulation‐considered in vitro cell cultivation has been recognized to be important. Moreover, this dynamic culture potentially offers two benefits: 1) fluid shear stress to promote cell–cell interactions and induction of differentiation and 2) randomized gravitational forces that affect intracellular signal transduction and gene expression.^[^ ^12^ ^]^

As a representative dynamic culture system, perfusion bioreactor has been utilized, even in clinical settings for immune cell expansion;^[^ ^13^ ^]^ they have specifically been used to control laminar flow (e.g., at a rate of 1.5 mL min^−1^) in order to supply, in a consistent manner, nutrients as well as gas and mechanical stimulation, and in this way achieve ex vivo mass production of functional immune cells.^[^ ^11^ ^]^ In addition, a recent study reported that fluid flow (1–4.27 mL min^−1^) enhances the vascularization of developing kidney organoids during nephrogenesis.^[^ ^14^ ^]^ While these systems are superior in dynamic cultures and in their capabilities of achieving mass production of functional cell products, their use is limited because they require an artificial filter membrane for retention of the cells and bulky external equipment such as peristaltic or syringe pumps and tubings. Agitation using a magnetic stirrer is also utilized in the dynamic cell culture,^[^ ^15^ ^]^ but inevitable contacts between the stir bar and cells might cause unwanted deteriorated cell viability and potential contamination.

Lab‐on‐a‐chip technology is appealing since the platform enables the precise control of microliter volumes of fluid containing microparticles in a highly miniaturized system. In microsystems where the Reynolds number is typically very small (Re  ≪  1), various types of active forces, e.g., pneumatic pressure, magnetic forces, and dielectric forces, have been employed to enhance the efficiency of mixing and/or separation.^[^ ^16^ ^]^ Meanwhile, emerging surface acoustic wave (SAW)‐based microfluidic technology has been considered an attractive alternative to the prevailing methods used for active fluid/particle control.^[^ ^17^ ^]^ Acoustofluidic platforms function by generating mechanical interactions between SAWs and adjacent liquid, and numerous such platforms have been produced and demonstrated the ability to separate microparticles, actuate and mix fluids, and modulate droplets.^[^ ^18^, ^19^, ^20^ ^]^ Notably, the SAW technology can also be advantageously used in biological assays, since it allows for an on‐demand controlled actuation in a label‐free manner, as shown by its use in the formation of multiple cell clusters, cell alignment in hydrogels, and cell sorting.^[^ ^21^, ^22^, ^23^, ^24^, ^25^, ^26^ ^]^ Although acoustic exposure is capable of causing cell damages due to heating or cavitation effects, a viability‐optimized acoustic manipulation can be biocompatible without any apparent negative impact on cellular state.^[^ ^27^, ^28^ ^]^

While many biological applications have benefited from the use of the SAW technology, most researchers have focused on producing cell displacements by exposing the cells to just short durations (seconds to minutes) of acoustic force. Only a few acoustofluidic systems have been reported to involve SAW‐derived effects on cells for longer durations (hours to days). The Cecchini group showed that 48 h of SAW‐derived acoustic streaming flow (ASF) in culture medium stimulated the proliferation of U‐937 cells.^[^ ^29^ ^]^ The Westerhausen group reported that 48 h of SAW irradiation did not result in severe damage to primary neuron cells.^[^ ^30^ ^]^ Meanwhile, the Guo group showed human brain organoid culture realized by exposing the cells to acoustofluidic forces for days with reinforcement learning‐based rotational control, but contacts between a plate and culture media containing the organoids had to be made.^[^ ^31^ ^]^ Thus, while a few studies have shown a lack of damage to cells resulting from their being exposed for a long time to SAWs, the biomechanical effects of the SAWs on the functions of the cells have not yet been investigated in a quantitative manner. Above all, the influence of SAW‐derived shear stress on immune cells has not yet been fully elucidated and warrants a more detailed investigation.

The objective of the present study was to investigate the functional responses of immune cells (NK‐92) being stimulated by traveling SAWs. Toward this end, a SAW‐based microreactor for dynamic cell culture was produced. This microreactor enabled us to apply fluid shear stress of 46.7 dyne cm^−2^ to the immune cells. Several parameters of the SAW system, e.g., total duration, frequency, applied voltage, and pulse repetition period and frequency of duty cycles, were adjusted to deliver a sufficient level of shear stress while minimizing increases in temperature. We analyzed several features of the system; for example, computational simulation and experimental flow visualization were carried out to provide a mechanical characterization of SAW‐induced ASF, and a biochemical investigation of cell vitality was performed, specifically of cell viability, proliferation and metabolism. Also, a fluorescence imaging investigation found greater levels of intracellular Ca^2+^ in SAW‐stimulated NK‐92 cell aggregates than in the static culture. Finally, an addition of protein kinase C (PKC) activator to the SAW microreactor significantly augmented the cytotoxic functions of the NK‐92 cells against target K‐562 cells, including having increased the relative LAMP‐1 expression levels and cell‐mediated target cell lysis.

## 2 Results and Discussion

### 2.1 Development of SAW Microreactor

**Figure 1:**
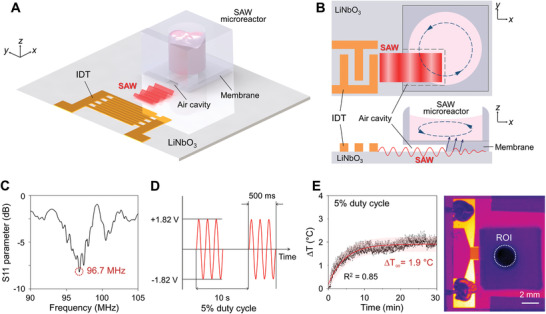
SAW‐based microreactor system for dynamic cell culture. A,B) Schematics illustrate the working mechanism of the SAW microreactor. After being generated from IDT electrodes, the SAWs propagate along a LiNbO_3_ substrate and refract into a fluid in the microreactor. The attenuation of SAWs in the fluid creates a time‐averaged body force that pushes the fluid, eventually generating ASF inside the microreactor. C) Power reflection spectrum of a fabricated IDT indicates a peak frequency of 96.7 MHz. D) Experimental SAW parameters are settled as follows, 3.64 V_p‐p_ and a 5% duty cycle (500 ms‐on and 9500 ms‐off). E) Temperature elevation measurements with an infrared camera show a temperature increase of 1.9 °C at the SAW parameters. The red line and pink band in (E) represent fitted exponential curve and 95% prediction band of measurements, respectively.

We developed a microreactor system including an open‐top polydimethylsiloxane (PDMS) well on a lithium niobate (LiNbO_3_) substrate where cascades of acoustic energy transfer exist, namely, propagation of interdigital transducer (IDT)‐generating SAWs along the substrate, entry into acoustic cavity and encounter with PDMS membrane/fluid by the SAWs, conversion of refracted SAWs into longitudinal waves in liquid media of the PDMS well, and formation of ASF derived from attenuation (**Figure** **1**A,B; Figure S1,S2, Supporting Information). The ASF consequently applies dynamic shear stress to the suspended immune cells. We expected that the net fluid motion inside the microreactor created by SAWs would be beneficial for cell culture due to this motion applying mechanical stimulation and helping to deliver nutrients to cells. In general, in the culture media of PDMS wells, the dynamic behavior of the ASF is highly affected by the attenuation length of the substrate. Given the focusing effects of standing SAWs on nodal positions by the standing SAWs, in the current work we designed the electrode spacing (*λ*_SAW_/4) of the IDTs to be 10 µm in order for the corresponding attenuation length (0.483 mm) to be shorter than the 3 mm diameter of the PDMS well. This design was included to prevent the development of standing SAWs that otherwise would have resulted from reflections of propagating sound waves from the substrate at the PDMS wall.^[^ ^17^ ^]^ The peak frequency of the fabricated IDT was confirmed, using a vector network analyzer, to be 96.7 MHz (Figure 1C; Figure S3, Supporting Information), and hence the corresponding attenuation length of the sound waves met the criterion of being shorter than the diameter of the PDMS well.

During refraction of SAWs from a piezoelectric substrate to liquid media, the acoustic energy was attenuated due to absorption of SAW by the viscoelastic PDMS membrane, resulting in the partial conversion of the energy to heat.^[^ ^27^ ^]^ In our work, we monitored the heat generated by the SAWs, due to such heat possibly causing critical damage to the cells. We set various duty cycle conditions such as SAW‐off periods from 2833 to 29 500 ms with a fixed SAW‐on period of 500 ms (Figure 1D). Under SAW treatments at two levels of power (3.64 and 2.84 V_p‐p_) and six different duty cycles, the increase in temperature of a PDMS well containing distilled water was recorded using an infrared camera (Figure S4, Supporting Information). As expected, longer SAW‐off periods resulted in less of a temperature increase at each of the tested power levels, specifically down from ∆3.1 °C (2833 ms SAW‐off) to ∆0.93 °C (29 500 ms SAW‐off) at 3.64 V_p‐p_, and from ∆2.1 °C (2833 ms SAW‐off) to ∆0.51 °C (29 500 ms SAW‐off) at 2.84 V_p‐p_. Based on the results, we settled on conditions including a 5% duty cycle with repeating steps of 9500 ms SAW‐off and 500 ms SAW‐on periods at 3.64 V_p‐p_; these conditions yielded a temperature increase of 1.9 °C (Figure 1E). The conditions were determined based on balancing concerns regarding temperature elevation and sufficient shear stress to NK cell aggregates. Indeed, while a stable temperature is desired for the physiological function of the cell, the criteria for temperature increases up to 3 °C have been accepted.^[^ ^32^, ^33^ ^]^ To correct the temperature increases (1.9 °C) for the SAW‐conditioned group, we set the temperature of the cell culture incubator as 35 °C.

### 2.2 Characterization of ASF in the SAW Microreactor

**Figure 2:**
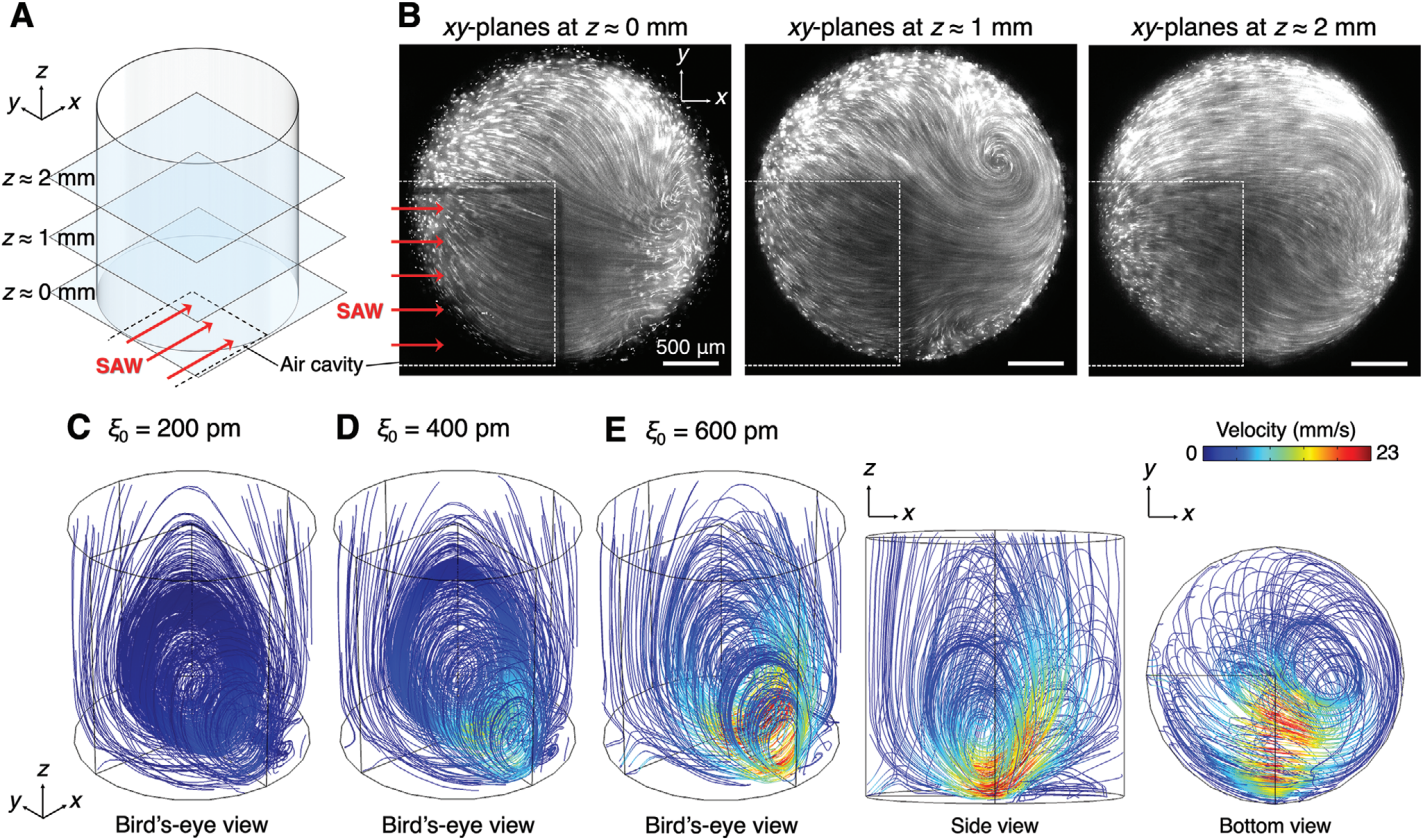
SAW‐derived ASF in the microreactor. A) ASF in the SAW microreactor having a cylindrical domain of 3 mm diameter and 3 mm height is characterized. B) Representative images show a visualization of fluorescence microparticles at three *xy*‐planes, specifically at *z* ≈ 0, 1, and 2 mm from the bottom PDMS membrane. The shown images are 10‐stacked‐images in respective *xy*‐planes. All scale bars 500 µm. C–E) Time‐averaged streamlines at varying initial displacements (*ξ*_0_ = 200, 400, and 600 pm) are shown.

The proposed acoustofluidic method to stimulate the immune cells suspended in a microreactor is based on the traveling SAW‐induced ASF. We conducted experimental and numerical flow visualization of the SAW‐induced ASF formed within the microreactor for thorough characterization. First, we explored the flow patterns of fluorescently labeled microparticles by varying locations and shapes of the acoustic cavity (Figure S5, Supporting Information). We finally selected a quarter acoustic cavity to pursue one‐directional flow rather than two‐directional flow being observed in the middle and side cavities; one was directed to outside of Rayleigh angle axis and the other was directed to inside of the axis called an anechoic corner.^[^ ^34^ ^]^ A steady 3D vortical flow formed in the microreactor under continuous SAW excitation of 96.7 MHz at 3.64 V_p‐p_ was visualized in the form of the tracing particle path lines at three different depths of *z* ≈ 0, 1, and 2 mm (**Figure** **2**A,B; Movie S1, Supporting Information). At the bottom‐plane (*z* ≈ 0 mm), the tracer particles traveled primarily at 45° to the +*x* axis in the *xy*‐plane due to the partial exposure to the +*x*‐propagating SAWs and resulting longitudinal waves, as well as the geometrical confinement of the circular microreactor. Through the particle image velocimetry (PIV) analysis, we obtained 2D maximum PIV velocity (*V*_2D_) at the bottom‐plane to be 7 mm s^−1^ (Figure S6, Supporting Information). Accordingly, given the SAW refraction equation, *V*_3D_ ≈ *V*_2D_/sin *θ*_R_ (where the Rayleigh angle *θ*_R_ ≈ 22°), the 3D maximum flow velocity (*V*_3D_) was calculated as 19 mm s^−1^.^[^ ^29^ ^]^ At the *xy*‐plane at *z* ≈ 1 and 2 mm, the ASF‐induced rotating flow was clearly visualized with the center of the vortical flow gradually shifted in the *y*‐direction with increasing *z*. Despite the experimental observation based on the 2D microscopic images, we confirmed the 3D vortical flow in the portion of ASF formed within the microreactor and estimated the 3D ASF velocity as 19 mm s^−1^.

For 3D flow visualization and in‐depth investigation, we performed computational analysis of the 3D vortical flow induced by the continuous SAW excitation to the fluid within the microreactor (Figure S7, Supporting Information). The continuity equation was numerically solved together with the Navier‐Stokes equation with the time‐averaged nonlinear acoustic streaming body force as a body force to obtain the 3D flow field in the microreactor. Under the steady‐state conditions, the particle pathlines obtained in the experimental flow visualization coincide with the ASF streamlines. The ASF velocity increased with increasing the initial displacement (*ξ*_0_), which is proportional to the voltage applied to the IDT, as clearly confirmed in the obtained ASF streamlines results at *ξ*_0_ = 200, 400, and 600 pm (Figure 2C–E). The initial displacement value of 600 pm was empirically chosen to achieve the maximum ASF velocity of approximately 23 mm s^−1^. The asymmetrically applied SAWs at the bottom of the microreactor in the *x*‐direction refracted at the Rayleigh angle of approximately 22° in the *xz*‐plane and gradually attenuated in the fluid due to viscous damping, resulting in the SAW‐induced ASF in the form of the 3D vortical flow. The particle tracing‐based experimental observation at varying *xy*‐planes was found to be in good agreement with the numerically obtained 3D velocity field. Based on the flow field, the trajectories of the particles that served as virtual immune cells were traced by considering the flow‐induced drag force, gravitational force, and acoustic streaming body force. For simplicity, the immune cells were regarded as solid microspheres with the same density (1060 kg m^−3^) and diameter (14.1 µm in Figure S8 in the Supporting Information). In the particle tracing simulation, total numbers of 273 particles were initially distributed in three different layers at *z* = 1, 1.5, and 2 mm. The virtual immune cell trajectories were numerically visualized at varying initial displacements (Movie S2, Supporting Information). Notably, the maximum shear stress applied to the cells was computed as 46.7 dyne cm^−2^ at the bottom plane of the PDMS well (Figure S9, Supporting Information),^[^ ^35^ ^]^ which is consistent with the previous observations for cellular alteration (i.e., Piezo1 activation and Ca^2+^ influx).^[^ ^36^, ^37^ ^]^ The discrepancy for the 3D maximum flow velocities between the experimental and computational analysis can be attributed to inaccurate 2D PIV calculation and different dynamic viscosities of fluid.

### 2.3 Vitality of Cells Subjected to the SAW Microreactor

The use of the SAW microreactor system may adversely affect the vitality of suspended cells.^[^ ^32^, ^38^ ^]^ Specifically, some of the energy of refracted acoustic waves is dissipated as thermal energy, and the generated heat may adversely affect the growth of cells. In addition, excessive shear stress from the ASF could damage cells. In the current work, we first set out to provide SAW conditions that would not adversely affect the vitality of the cells used. Based on the aforementioned thermal characterization, we set the SAW duty cycle and power to 5% (with repeats of 500 ms SAW‐on and 9500 ms SAW‐off) and 3.64 V_p‐p_, respectively. We also adjusted the initial temperature of the cell culture chamber to 35 °C in consideration that, with SAW‐generated heat, it would reach as high as 37 °C.

**Figure 3:**
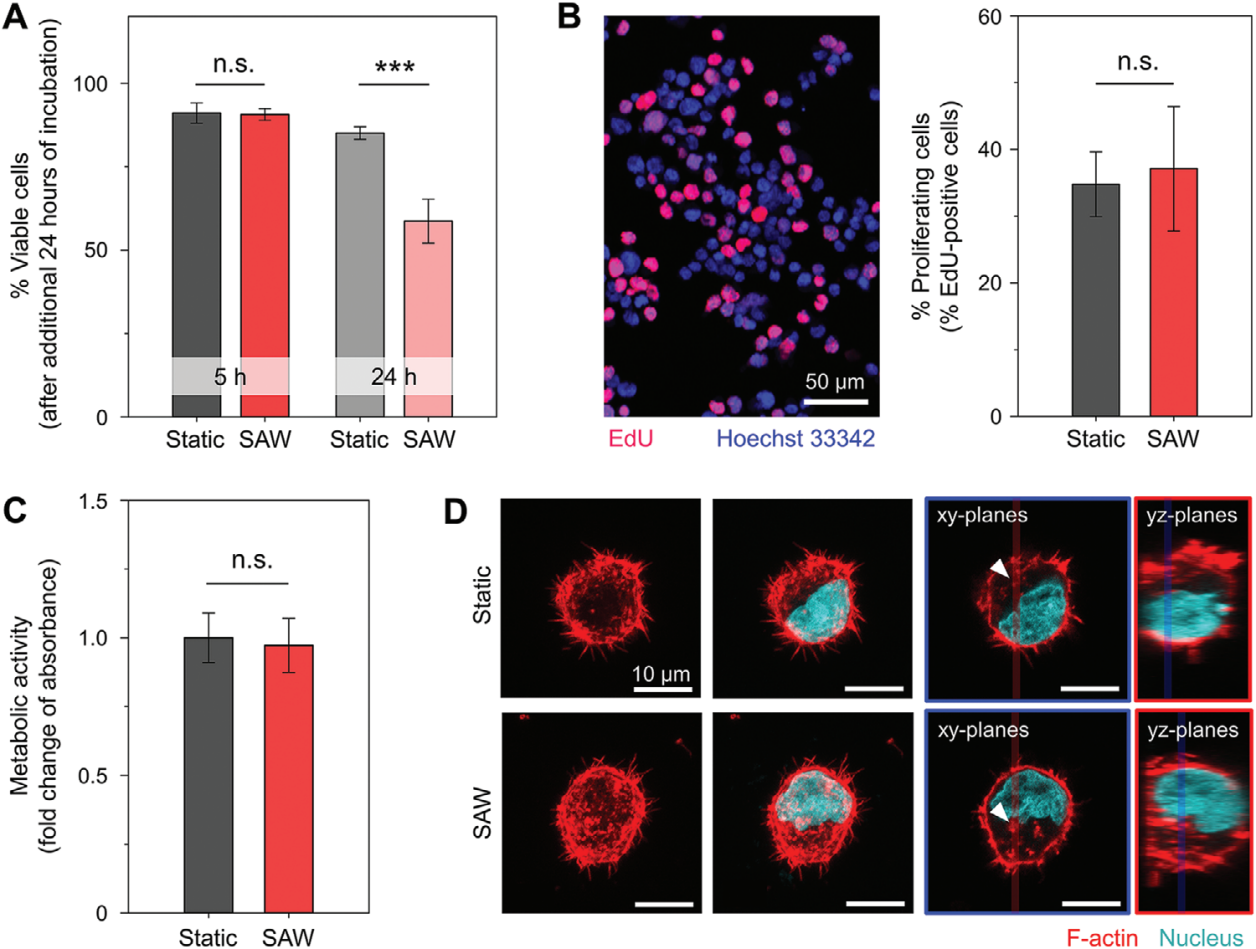
NK‐92 cell vitality under selected SAW conditions. A) Trypan blue exclusion assays were used to show the percent viable cells under 5 and 24 h with and without SAW and the SAW condition did not result in any significant change of cell viability when applied for 5 h (*n* = 5–6 chips). B) Fluorescence staining of EdU incorporation into cell nuclei indicates no suppression in cell proliferation under the SAW condition (*n* = 4 chips). C) Metabolic activities of conditioned cells are measured based on the absorbance changes of media and the results show no detrimental effects by the SAW treatment (*n* = 6 chips). D) F‐actin fluorescence staining shows no destruction of cytoskeletal structures of the conditioned cells. All scale bars 10 µm.

To assess the effects of SAWs on the vitality of the cells, we first performed a Trypan blue exclusion test against the NK‐92 cells. For this test, the cells were first exposed to SAWs for 5 or 24 h and further left at 37 °C 5% CO_2_ for 24 h. Then the cells were mixed with a Trypan blue solution, and the numbers of live and dead cells were immediately counted with a brightfield microscope. The percentage of cells exposed to 5 h of SAWs that were indicated to be viable was about 90.6 ± 1.8% (**Figure** **3**A). But this percentage for those cells exposed to 24 h of SAWs decreased to 58.7 ± 6.6%, which was also considerably lower than the corresponding 85.1 ± 1.9% value for the static condition, i.e., when no SAWs were applied (*p* < 0.001) (Figure 3A; Figure S10, Supporting Information). Thus, the longer 24 h duration of exposure to SAWs was concluded to damage the cells. Next, we performed a 5‐ethynyl‐2′‐deoxyuridine (EdU) incorporation assay to investigate the effect of the SAW treatment on DNA synthesis. After labeling cells with EdU and fluorescent dyes, a confocal fluorescence microscope was used to identify proliferating cells, i.e., those appearing as both red and blue, among the harvested cells, i.e., those appearing as blue (Figure 3B; Figure S10, Supporting Information). The results of counting these types of cells showed that the percentage of total cells that were proliferating cells was not significantly different (34.7 ± 4.8% vs 37.1 ± 9.3%, *p* > 0.05) between the static and 5 h SAW conditions (Figure 3B). In addition, we monitored cellular metabolic activity by using a resazurin‐based reagent. Here, the conversion of resazurin to resorufin by live cells was monitored by measuring the resulting pronounced color change in the culture media surrounding the cells using absorbance‐based plate readers. No significant difference in absorbance was observed between 5 h of exposure to SAWs and the static condition (Figure 3C). Finally, we carried out a fluorescence staining of F‐actin in the NK‐92 cells to determine whether SAWs disrupted the cytoskeletal structure of the cells. Observations with a confocal microscope did not indicate the occurrence of any specific damage to the F‐actin inside the NK‐92 cells regardless of SAW treatment for 5 h (Figure 3D; Figure S10, Supporting Information).

### 2.4 Calcium Influx to Suspended Immune Cells under SAW‐Derived Fluid Shear Stress

**Figure 4:**
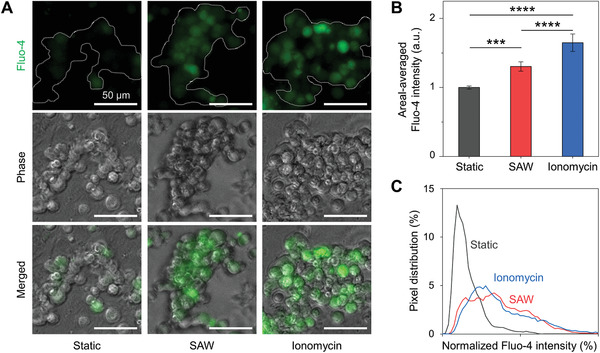
Ca^2+^ influx to SAW‐treated NK‐92 cells. A) Epifluorescence microscope images show Fluo‐4 Ca^2+^ indicating dyes in NK‐92 cell aggregates under static, SAW, and ionomycin for 30 min. All scale bars 50 µm. B) Areal‐averaged Fluo‐4 intensities are quantified from the cell aggregates (>5 cells) of fluorescence images to evaluate Ca^2+^ influx to the cells. The results show increased Ca^2+^ influx to SAW‐conditioned NK‐92 cell aggregates compared to that of static condition (*n* = 6 chips), which is lower than that of ionomycin condition as a positive control. C) Pixel distributions are analyzed for the cell aggregates shown in (A). Both SAW and ionomycin conditions have similar curves where more pixels are concentrated in the higher Fluo‐4 intensity ranges.

We aimed to investigate the effect of SAW‐derived ASF on Ca^2+^ influx into the NK‐92 cells. We applied SAWs to the suspended NK‐92 cells in the microreactor for 30 min with a 5% duty cycle at 3.64 V_p‐p_. Using a Fluo‐4 Ca^2+^ indicator, which in general exhibits a large increase in fluorescence intensity (>100‐fold) upon binding Ca^2+^, we observed changes in intracellular Ca^2+^ concentrations of the NK‐92 cell aggregates upon their exposure to SAWs (**Figure** **4**A). Greater Fluo‐4 fluorescence was observed, using the epifluorescence microscope, in the NK cell aggregates exposed to SAWs than in the static condition. The areal‐averaged Fluo‐4 intensity, a metric calculated by dividing the sum of the Fluo‐4 intensity by the area of the cell aggregates, was obtained for each condition from the microscope images to compare the intracellular Ca^2+^ between conditions (Figure 4B). For the static, SAW, and ionomycin (Ca^2+^ ionophore) conditions, the areal‐averaged Fluo‐4 intensity values were 1.00 ± 0.02, 1.30 ± 0.07, and 1.65 ± 0.13, respectively (Figure 4B). From the cell aggregates of the nonstatic conditions, i.e., the SAW and ionomycin conditions, more pixels were concentrated in the higher Fluo‐4 intensity ranges (Figure 4C), supporting the previous result. Mechanical stress has been shown to increase the concentration of intracellular Ca^2+^ by affecting mechanosensitive ion channels (e.g., TRPC and Piezo1).^[^ ^39^, ^40^, ^41^, ^42^ ^]^ Importantly, acoustic pressure has been shown to be able to affect Ca^2+^ influx into neuronal,^[^ ^43^ ^]^ endothelial,^[^ ^44^ ^]^ epithelial,^[^ ^45^ ^]^ osteoblastic,^[^ ^46^ ^]^ and cancer cells.^[^ ^47^, ^48^ ^]^ Acoustical streaming has also been reported to elicit Piezo1 activation and intracellular Ca^2+^ response,^[^ ^36^, ^37^ ^]^ consistent with our results.

### 2.5 Enhanced Lysosomal Marker Expression and Target Cell Lysis under Cooperation of SAWs and PMA

**Figure 5:**
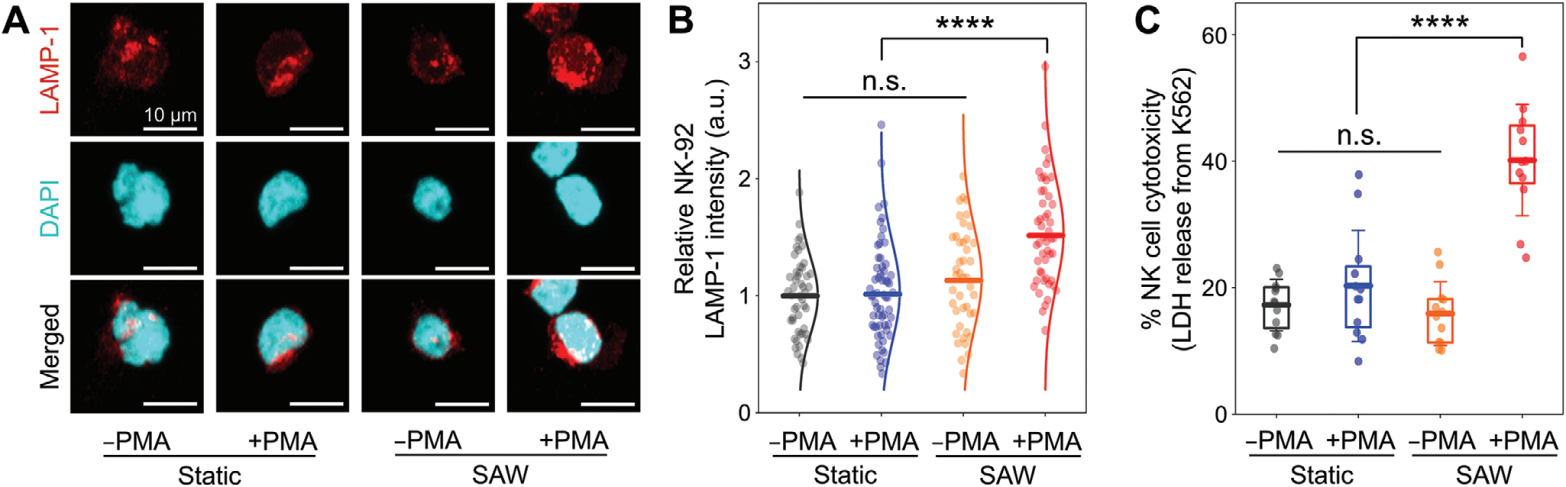
The LAMP‐1 expression levels of NK‐92 effector cells and their cell‐mediated cytotoxicity against target K‐562 cells. A) Representative immunofluorescence images show the LAMP‐1 expression of NK‐92 cells in the presence or absence of SAWs and/or PMA. All scale bars 10 µm. B) Relative LAMP‐1 intensity of NK‐92 cells is shown for the four indicated conditions. Each dot indicates the quantified intensity of respective cells of the conditions (*n* = 43 from four chips). C) LDH releases from K‐562 cells to measure the NK cell cytotoxicity are quantified for the four indicated conditions. Each dot indicates the quantified values of respective chips of the conditions (*n* = 12 chips). The edges of the boxes are the 25th and 75th percentiles and the whiskers are standard deviations.

NK cell cytotoxicity has been recognized as a stepwise and highly regulated process; central to this process is the initial contact between NK and target cells, immunological synapse formation, cytotoxic granule secretion to target cells, and target cell apoptosis.^[^ ^49^, ^50^ ^]^ As cytotoxicity of lymphocytes has been strongly dependent on Ca^2+^ influx,^[^ ^51^, ^52^ ^]^ we further examined whether the SAW‐induced elevation of intracellular Ca^2+^ levels leads to the enhancement of NK cell degranulation and cell‐mediated anticancer cytotoxicity. For this experiment, NK‐92 effector cells in the microreactor were exposed to SAWs with a 5% duty cycle at 3.64 V_p‐p_. Their relative LAMP‐1 (an important lysosomal marker^[^ ^53^ ^]^) expression levels were analyzed with a fluorophore‐conjugated antibody and a confocal microscope (**Figure** **5**A). Unexpectedly, SAW treatment alone for 3 h was insufficient to induce a significant increase in relative LAMP‐1 intensity compared to that of the static culture (black vs orange in Figure 5B). NK‐92 cells treated simultaneously with SAWs and phorbol 12‐myristate 13‐acetate (PMA; an analogue of the diacylglycerol (DAG) to activate the signal transduction of PKC), however, did show a significant increase in LAMP‐1 intensity, specifically a 1.54 ± 0.46‐fold increase, compared to the LAMP‐1 intensities of the other conditions including the static culture (*p* < 0.001) (red vs others in Figure 5B).

Next, to investigate cell‐mediated cytotoxicity, NK‐92 cells treated with SAWs for 5 h were cocultured with K‐562 target tumor cells in a static environment for 4 h (E:T ratio of 5:1). After coculture, NK‐92 cells’ cytotoxicity was evaluated by measuring lactate dehydrogenase (LDH) activity in culture supernatants, which is based on the conversion of lactate to pyruvate in the presence of LDH that is released from damaged K‐562 cells.^[^ ^54^ ^]^ Similar to the LAMP‐1 results, including either SAWs or PMA alone had an insignificant effect on NK cell cytotoxicity (black vs blue or orange in Figure 5C). Only the cotreatment of PMA and SAWs resulted in a significant increase of NK cell cytotoxicity, in fact a quite marked increase to 40.1 ± 8.8% (*p* < 0.001) (red vs others in Figure 5C).

The NK cell cytotoxicity thus did not result from an SAW‐derived Ca^2+^ influx alone but was improved by the addition of PMA to the SAW microreactor.^[^ ^55^ ^]^ For instance, the expression of lysosomal marker LAMP‐1 of NK cells was enhanced for cells simultaneously treated with PMA and the Ca^2+^ ionophore.^[^ ^56^, ^57^, ^58^ ^]^ Also, PKC activation and an increase in Ca^2+^ together triggered degranulation in mast cells.^[^ ^59^ ^]^ Despite NK cells requiring Ca^2+^ to regulate cellular immune functions (e.g., immune synapse formation, cytokine production and cytotoxic activity), incubation with Ca^2+^ ionophore alone did not enhance cell‐mediated NK cell cytotoxicity.^[^ ^60^ ^]^ Similarly, in a T cell, the Piezo1‐driven Ca^2+^ influx was reported to lead to cell activation in the presence of soluble CD3/CD28‐based stimulation.^[^ ^6^ ^]^ Meanwhile, sole inclusion of PMA was insufficient to trigger cytotoxic functions as well, resulting in compromised cell‐mediated cytotoxicity.^[^ ^61^, ^62^ ^]^ With the supporting observations, our results indicate that copresence of the PKC activator PMA and an increased Ca^2+^ ion concentration resulting from SAW mechanical stimulus yielded enhancements of granule protein expression and target cell lysis of NK‐92 cells.

The signaling transduction pathways of NK cell activation is a highly sophisticated process, which is known to be dependent on the phospholipase C *γ* (PLC*γ*) family of proteins.^[^ ^63^ ^]^ Among them, PLC*γ*1 and PLC*γ*2 are cytoplasmic enzymes that catalyze the cleavage of membrane‐associated phosphatidylinositol 4,5‐bisphosphate (PIP_2_) into inositol triphosphate (IP_3_) and DAG. IP_3_ stimulates the release of Ca^2+^ from endoplasmic reticulum and DAG activates protein such as PKC, which are two indispensable transductions that we here substituted with SAW‐derived Ca^2+^ influx and DAG analogue PMA, respectively (Figure S11, Supporting Information). The details for regulatory mechanism that controls NK cell activation have not been elucidated to date, and the effects of SAW on Ca^2+^ influx and cytotoxicity also remain to be explored further. Nevertheless, these results showed that the acoustic pressure and resulting fluid shear stress could be partially involved in the NK cell activation as mechanical cues, in addition to the existing biochemical stimuli.^[^ ^9^ ^]^

Recently, Neild group fabricated a system that facilitates the assessment of cellular behaviors (e.g., adhesion, morphology, and mortality) following acoustic exposure.^[^ ^64^ ^]^ Flowing cells in the microchannel were treated with SAW excitation for about 19 seconds, and the phenotypic analysis revealed different responses across the cellular types (MSCs; sensitive, L929 and MG63; intermediate, HaCaT; resistant). Notably, the acoustic stimulation under a threshold increased the cellular metabolic activity by mitigating or overriding the impaired activity that conferred by flow condition, consequently resulting in the recovery of the metabolism to their original status. They hypothesized that those results may be attributed to type‐specific variations in cell stiffness, which provides susceptibility or resistance to the external mechanical forces. Compared to the recent research, we examined the NK‐92 cells’ changes after SAW exposure. Under the repetitive pulses of acoustic stimulation for 5 h, the NK‐92 cells maintained their health status in terms of viability, proliferation, metabolic activity, and cytoskeletal structures. However, the extension of SAW treatment to 24 h significantly reduced the viable NK‐92 cells to 58.7 ± 6.6%, and we anticipate that this extension could contribute to other cellular functions for detrimental states as well. In addition to cellular vitality, we investigated Ca^2+^ influx and cytotoxic functions of NK‐92 cells after SAW stimulation. We hypothesize that the elevated Ca^2+^ influx may be attributed to the ASF‐derived shear stress via activation of Ca^2+^ ion channels (i.e., TRPC and Piezo1) or pore formation at the cell membrane (i.e., sonoporation),^[^ ^36^, ^37^, ^65^ ^]^ which requires further investigation (Figure S11, Supporting Information).

## 3 Conclusions

We investigated cellular responses to the application of traveling SAWs in a microreactor. While shaping of in vitro NK cell activity with biochemical stimuli (e.g., cytokines, additives, autologous accessory cells, and autologous or allogeneic feeder cells) has been extensively studied, this acoustic‐based microreactor can pave the novel avenue in dynamic cell culture for more functional immune cells. Our dynamic cell culture using SAWs was used with the aim of altering the functions of NK‐92 cells but is generally difficult to achieve in microsystems due to their small Reynolds numbers. This technique is dissimilar from the previously reported methods of agitating cell culture media, namely, perfusion bioreactor with membrane filters and magnetic stirrer. The acoustofluidic microreactor developed in the current work was miniaturized, disposable and biocompatible, and even enabled dynamic cell cultures to be set up. The SAWs produced by an IDT propagated along a LiNbO_3_ substrate and refracted into the media above a PDMS membrane, resulting in a contact‐free and rotor‐free achievement of a chaotic ASF in the microreactor. The ASF affected the entire microreactor with a maximum flow velocity of 23 mm s^−1^ and corresponding fluid shear stress of 46.7 dyne cm^−2^. Fluorescence imaging using 2 µm diameter microbeads allowed for a visualization of the ASF in the microreactor, and showed that 3D agitation occurred throughout the media. Before investigating the effects of SAWs on cell function, we set out to determine experimental parameters for the SAW system that would be adequate at protecting the cells from being damaged by excessive heat and flow rates resulting from the applied acoustic energy. The best SAW parameters in this regard were determined to be 5% duty cycle (repetitive steps of 500 ms SAW‐on and 9500 ms SAW‐off) at 3.64 V_p‐p_ for 5 h, and with these conditions, we found that the viability, proliferation, metabolism, and cytoskeletal structures of NK‐92 cells remained unchanged regardless of SAW treatment. From fluorescence analysis of Fluo‐4 Ca^2+^ dye, markedly higher intracellular Ca^2+^ concentrations were observed for SAW‐treated cells than for the static culture. The SAW‐triggered Ca^2+^ influx, however, by itself resulted neither in an enhancement of relative LAMP‐1 expression levels in the NK‐92 cells nor in an enhancement of lysis of target cells by the NK‐92 cells. But the addition of PKC activator PMA to the SAW microreactor did enhance the cytotoxic functions of NK‐92 cells in terms of both lysosomal marker LAMP‐1 and target K‐562 cell lysis. The combination of Ca^2+^ influx derived from SAWs and the inclusion of the PKC promoter seemed to result in activation of cytotoxicity‐related signaling pathways of NK‐92 cells. The systematic characterizations and experimental results of the current work confirmed the utility of our SAW‐based microreactor for dynamic cell culture. This microsystem could be used to complement the available bulky platforms while maintaining the performance of agitating culture media, highlighting the potential of a novel way to achieve advanced cell culture applications and its implications for the study of mechanosensitive immune responses.

## 4 Experimental Section

### Materials and Cells

Materials were purchased from manufacturers as follows: alpha minimum essential media (*α*MEM) without phenol red, 100X GlutaMax, 1 m HEPES, 100X nonessential amino acid (NEAA), fetal bovine serum (FBS), and horse serum were purchased from Gibco. Penicillin/streptomycin, Pluronic F‐127, Hanks’ balanced salt solution (HBSS) with Ca^2+^ and Mg^2+^, a Click‐iT EdU imaging kit, and a CAS‐block histochemical reagent were purchased from Thermo Fisher Scientific. A Fluo‐4 direct calcium assay kit, Hoechst 33342, Pluronic F‐127, 4′,6‐diamidino‐2‐phenylindole (DAPI), Trypan blue, Prolong Glass Antifade Mountant, Rhodamine Phalloidin, and PrestoBlue were obtained from Invitrogen. PMA, Cytotoxicity Detection Kit Plus, bovine serum albumin (BSA), a poly‐l‐lysine solution, and trichloro (1H,1H,2H,2H‐perfluorooctyl) silane were purchased from Sigma Aldrich. Cy3‐conjugated LAMP‐1 antibody was purchased from Abcam, recombinant human interleukin‐2 (IL‐2) from PeproTech, ionomycin from EMD Millipore, paraformaldehyde (PFA) from Biosesang, and PDMS (Sylgard 184 A/B) from Dow Corning.

NK‐92 cells (ATCC), a well‐characterized human NK cell line that has demonstrated promising anticancer activities in clinical trials,^[^ ^66^ ^]^ were cultured in a complete media (*α*MEM supplemented with 12.5% v/v FBS, 12.5% v/v horse serum, 1% v/v GlutaMax, 1% v/v NEAA, 20 × 10^−3^ m HEPES, and 100 U/mL IL‐2). K‐562 cells (Korean Cell Line Bank), which are cells of a human erythroleukemic cell line, were cultured in RPMI1640 supplemented with heat‐inactivated 10% v/v FBS. All media contained penicillin/streptomycin at a final concentration of 1%. All cells were maintained in a 37 °C 5% CO_2_ incubator except when subjected to SAWs.

### Acoustofluidic Device Configuration

An acoustofluidic device included a disposable PDMS well and a straight‐finger IDT deposited onto a piezoelectric LiNbO_3_ substrate. The IDTs were fabricated by patterning bimetallic electrodes (Cr/Au: 300 Å/1000 Å) onto the LiNbO_3_ substrate (MTI Korea) using an E‐beam evaporation process. The electrode spacing and width (*λ*_SAW_/4) of IDTs were designed to result in an IDT‐produced acoustic wavelength of 10 µm (corresponding target resonant frequencies of 100 MHz). Each IDT included a 1.5 mm length aperture and 20 pairs of fingers. The power reflection spectrum of the IDTs was analyzed with a vector network analyzer (E5071B, Agilent Technologies) together with an electronic calibration module (Ecal 85093C, Agilent Technologies), identifying the resonance frequencies of IDTs as 96.7 MHz.

The PDMS wells were made by bonding a PDMS membrane to a perforated PDMS block (Figure S1, Supporting Information).^[^ ^19^ ^]^ The PDMS membrane was fabricated with an air cavity having a width of 1.5 mm and a height of 40 µm on an SU‐8‐deposited silicon wafer by a conventional photolithography process.^[^ ^67^ ^]^ The air cavity reduced the attenuation of propagating SAW before the SAW encountered PDMS membrane/fluid. The wafer was treated with trichloro(1H,1H,2H,2H‐perfluorooctyl)silane for 30 min in a vacuum chamber and immediately coated with a PDMS mixture (base:curing ratio of 35:1) using a spin coater at 1400 rpm for 30 s. The PDMS membrane (thickness ≈ 75 µm) was cured at 65 °C for 2 h and then bonded with the PDMS block containing a 3 mm diameter hole, with this bonding made by carrying out an oxygen plasma treatment (Femto Science). The resulting PDMS well was sterilized with 70% ethyl alcohol and then placed on the LiNbO_3_ substrate; the well and substrate were completely held in contact with each other by van der Waals forces without additional chemical and/or physical bonding.^[^ ^68^ ^]^

### Working Mechanism of the SAW Microreactor

The working principle of the SAW microreactor can be illustrated as follows. The system was designed to have SAWs with a resonant frequency generated from the IDTs. The SAWs propagated along the substrate to encounter the PDMS well device, with the difference in speed of sound between the substrate, the PDMS membrane, and the cell media, causing some of the SAWs to refract into the cell media along the direction known as the Rayleigh angle, given by the equation *θ*_R_ *=* arcsin(*c*_f_ */c*_s_), where *c*_f_ and *c*_s_ indicate the acoustic wave velocities of the fluid and piezoelectric substrate, respectively. For the case of SAWs traveling from 128° Y‐cut X‐propagation LiNbO_3_ into fluid, the Rayleigh angle would be *θ*_R_ = 22° based on *c*_f_ = 1480 m s^−1^ and *c*_s_ = 3950 m s^−1^.^[^ ^18^ ^]^ The attenuation of the oscillating displacement in the fluid, induced by the refracted acoustic waves at the Rayleigh angle in the *xz*‐plane, creates a time‐averaged body force that accelerates the fluid in the direction of wave propagation, eventually generating an ASF inside the culture media. The cells in the microreactor are expected to experience shear stress from the SAW‐induced ASF.

Off‐chip cultured NK‐92 cells were washed with HBSS and adjusted to 300 000 cells mL^−1^ with *α*MEM (100 U mL^−1^ IL‐2), and a volume of 21 µL of the NK‐92 cells was introduced to the PDMS well on the LiNbO_3_ substrate. AC signals were produced by using an RF signal generator (N5181B‐501, Keysight Technologies) and amplified by using a power amplifier (ZHL‐100 W‐GAN+, Mini‐Circuits) powered by a DC power supply (UP‐3015, Keysight Technologies). The output signals were applied to the IDTs via cables to generate SAWs. The SAWs were repeatedly turned on for 500 ms and off for 9500 ms (5% duty cycle) for the indicated times. The peak frequency was 96.7 MHz and the V_p–p_ applied to the IDTs was 3.64 V.

### Heat Monitoring with Infrared Ray Camera

To detect heat generated during the SAW treatments, an infrared camera (A325sc, Teledyne FLIR) with a macro lens (FLIR Close‐Up Lens 1× (25 µm), Teledyne FLIR) was set to record the heat profile of the distilled water in the PDMS well. The setup was placed in a semiclosed chamber at room temperature. The SAWs were applied with the indicated conditions of duty cycles for 30 min, and the infrared images were captured at 60 frames min^−1^ with an emissivity of 0.95.

### Visualization of ASF Formed in the Acoustofluidic Microreactor and Estimation of the ASF Velocity

For the experimental flow visualization, 2 µm diameter fluorescent polystyrene microparticles (Duke Scientific) were used as flow tracers under an inverted microscope (IX‐73, Olympus) with an objective lens (UplanFL 4X, Olympus). A high‐speed camera (VEO710‐L, Phantom) was used to capture the particle images under volumetric illumination by a continuous laser (MGL‐F‐532, Changchun New Industries Optoelectronics Technology). For SAW actuation, an RF signal generator (N931A, Keysight) with an RF amplifier powered by a DC power supply (EX50‐24, ODA technology) was used. When the ASF was under a steady‐state condition (about 3 s after the SAW was applied), the particle flow images at three planes (*z* ≈ 0, 1, and 2 mm) were captured at 100 fps. For quantitative estimation of the ASF velocity in the microreactor, we first applied the 2D PIV.^[^ ^69^ ^]^ We utilized PIVlab to calculate the flow velocity at each plane by 2D cross‐correlation, using an interrogation window of 68 × 68 pixels^2^ with 50% overlap for a coarse grid and 32 × 32 pixels^2^ with 50% overlap for the refined grid system.^[^ ^70^ ^]^ The Stokes number (*St*), a metric of the particle response time (*τ*_p_) over the flow time scale (*τ*_f_), can be calculated as *St* = *τ*_p_/*τ*_f_ = [(1/18)*ρ*_p_ *d*_p_^2^/*μ*_f_]/[*R*/*V*_2D@_ *_z_*_≈0_] ≈ 0.11 × 10^−7^, where *ρ*_p_ is the density of the particles, *d*_p_ is the particle diameter, *μ*_f_ is the dynamic viscosity of the fluid, *R* is the radius of microwell, and *V*_2D_ *_@z≈_*_0_ is the maximum flow velocity at bottom plane obtained from the 2D PIV results (≈7 mm s^−1^) (see Table S1 in the Supporting Information for the parameters). The magnitude of *St* was small to be negligible, which indicates that the particle velocity could approximately represent flow velocity.

Computational fluid dynamics simulation was performed as follows. Under the assumption that the shear wave contribution is negligible due to the relatively low speed of sound in the fluid, the nonlinear acoustic body force (*F*_ASF_) induced at a point (*x*, *z*) can be expressed as *F*_ASF_ = *ρ*_f_ *βu*(*x*, *z*)^2^, where *ρ*_f_ is the fluid density, *β* is the acoustic attenuation coefficient in the fluid, and *u*(*x*, *z*) = *ωξ*(*x*, *z*) is the displacement velocity.^[^ ^71^, ^72^ ^]^ The coefficient *β* is defined as *β* = (4*μ*/3 + *μ'*)*ω* ^2^/*ρ*_f_ *c*_f_^3^ where *μ* and *μ*′ are the dynamic and bulk viscosities of the fluid, and *ω* is the angular frequency. The wave displacement is approximated as *ξ*(*x*, *z*) = *ξ*_0_(e^−^ *^*α*x+*α*z^* ^tan^ *^*θ*^* e^−^ *^*β*z^* ^sec^ *^*θ*^*), where *ξ*_0_ is the initial displacement of the acoustic waves, *α* is the acoustic attenuation length along with the interface between the piezoelectric substrate and the fluid, and *θ* is the Rayleigh angle. The coefficient *α* is defined as *α* = *ρ*_f_ *c*_f_ */ρ*_s_ *c*_s_ *λ*_s_, where *ρ*_s_ is the substrate density, and *λ*_s_ is the acoustic wavelength in the substrate. For the numerical simulation of the time‐dependent SAW‐induced ASF in the microreactor with its diameter and height of 3 mm, we solved the continuity equation and the Navier–Stokes equation with *F*_ASF_ as a body force by using COMSOL Multiphysics (version 5.6, COMSOL). In addition, the particle tracing was conducted to simulate the 3D cell trajectories formed by the SAW‐induced ASF with the assumption of cells to be 14.1 µm diameter solid microspheres. To calculate the fluid shear stress applied to a cell by SAW‐derived flow, the shear stress (SS) was computationally analyzed by applying the following equation; SS = (3/2)·(*μV*/*r*),^[^ ^35^ ^]^ where *μ* is the fluid dynamic viscosity at 37 °C, *r* is the particle radius, and *V* is the velocity magnitude of computational analysis result at 600 pm initial displacement which is empirically chosen for high similarity with experiment.

### Immunofluorescence Staining

Cells mixed with HBSS were deposited onto a poly‐l‐lysine‐coated coverglass by subjecting the mixture to centrifugation for 5 min at 300 xG. The cells were then fixed with PFA (4% w/v in PBS) for 15 min and permeabilized with saponin (0.5% w/v in PBS) for 10 min at room temperature. The permeabilized cells, after being washed with BSA‐supplemented PBS (0.1% w/v) twice, were blocked with CAS‐block for 15 min, then incubated with a Cy3‐conjugated primary LAMP‐1 antibody overnight at 4 °C, and then incubated with DAPI for 1 h at room temperature. For F‐actin staining, the permeabilized cells were incubated with Rhodamine Phalloidin for 1 h at room temperature. The cells were washed five times with BSA‐supplemented PBS solution after every antibody incubation. The cells on the coverglass were sealed with Prolong Glass Antifade Mountant and imaged using a confocal fluorescence microscope (LSM 880, Carl Zeiss).

### Cell Viability, Proliferation, and Metabolic Activity

A Trypan blue assay was performed to investigate cell viability. After the SAW treatment, the PDMS well was transferred to a 37 °C 5% CO_2_ incubator and further incubated for 24 h. The medium containing cells was then harvested, mixed with 0.4% Trypan blue with a 1:1 volume ratio, and injected into a C‐chip (Incyto). The cells were immediately imaged with a brightfield microscope and live (presented as white) and dead (presented as black) cells were counted from the images.

The ability of the cells to proliferate was determined using a Click‐iT EdU imaging kit according to the manufacturer's instructions. The SAW‐treated cells were further incubated with EdU (10 × 10^−6^ m in the complete media) for 2.5 h in the 37 °C 5% CO_2_ incubator to incorporate EdU into the DNA of the cells during active DNA synthesis. After being mixed with HBSS, the cells were deposited onto a poly‐l‐lysine‐coated coverglass by subjecting the mixture to centrifugation for 5 min at 300 xG, and the cells were fixed and permeabilized as described above. Then, the cells were stained with EdU detection buffer and Hoechst 33342 (5 µg mL^−1^) at room temperature for 1 h each, and the stained cells were imaged with a confocal fluorescence microscope. The dimensions of the regions of interest (ROIs) were 850.19 × 850.19 µm^2^. The percentage of total cells that were proliferating cells was calculated as follows: 100 × (the number of cells that were both EdU‐positive and Hoechst‐positive/the number of cells that were Hoechst‐positive).

The metabolic activity of cells was evaluated using the resazurin‐based PrestoBlue Cell Viability Reagent according to the manufacturer's instructions. Briefly, the SAW‐treated cells were further incubated with the reagent for 6.5 h in the 37 °C 5% CO_2_ incubator and the samples were transferred to a 384‐well microtiter plate. Their absorbance values were measured using a microplate spectrophotometer (Epoch, BioTek) at wavelengths of 570 and 600 nm (reference wavelength). The absorbance values at 570 nm were normalized to the values at 600 nm.

### Calcium Imaging

We also set out to image the Ca^2+^ influx resulting from the application of SAWs. Before applying the SAW treatment, 1.5 × 10^6^ cells mL^−1^ NK‐92 cells were incubated with 1 X Fluo‐4 calcium dye with 2.5 × 10^−3^ m probenecid and 0.02% F‐127 for 45 min at 37 °C 5% CO_2_ according to the manufacturers’ instruction. The concentration of cells was then adjusted to 300 000 cells mL^−1^ with the complete media; the adjustment was done without subjecting the cells to centrifugation and any of its potential effects on intracellular calcium flux. After 30 min of SAW treatment, the cells were immediately imaged with an epifluorescence microscope (Carl Zeiss) equipped with an objective lens (LD Plan‐Neofluar 20x/0.4 Korr Ph 2 M27). Cells without SAWs or with 10 µg mL^−1^ ionomycin were placed in the incubator for 30 min, as a negative control or positive control, respectively. To quantify Ca^2+^ influx to NK‐92 cell aggregates, a metric of areal‐averaged Fluo‐4 intensity was calculated by dividing the sum of the Fluo‐4 intensity by the area of the cell aggregates (>5 cells) with ImageJ software (NIH).

### NK Cell Cytotoxicity toward Target Tumor Cells

The cytotoxicity of NK cells toward target K‐562 cells was assessed by using a Cytotoxicity Detection Kit Plus to measure the amount of LDH released. The serum concentration of culture media was adjusted to 5% FBS to increase the sensitivity of the assay according to manufacturer's recommendation. Briefly, NK‐92 cells obtained from PDMS wells were treated with SAWs for 5 h. Then, 1.2 × 10^3^ target K‐562 cells were cocultured with SAW‐treated and/or PMA‐treated (50 ng mL^−1^) NK‐92 cells at an effector‐to‐target (E:T) ratio of 5:1 for 4 h in the PDMS microreactor. After transferring the conditioned media into a 96‐well‐plate, the changes of absorbance at a wavelength of 490 nm due to LDH released from K‐562 cells were measured using the microplate spectrophotometer. After subtracting the absorbance of blank media from the absorbance of conditioned media, specific cytotoxicity was calculated as follows: % cytotoxicity = 100 × [(experimental release − effector spontaneous release − target spontaneous release)/(target maximal release − target spontaneous release)].

### Statistical Analysis

All measurements are reported as averages ± standard deviations unless otherwise specified. Measurements were compared using the Student's two‐tailed *t*‐test when comparing two conditions or one‐way analysis of variance (ANOVA) with the Tukey multiple comparison test when applicable. Statistical analysis was performed using OriginPro Software (OriginLab). All tests yielding *p* < 0.05 were considered to be statistically significant: *, **, ***, and **** indicate *p* < 0.05, 0.01, 0.001, and 0.0001 between the conditions, respectively, and n.s. indicates statistically no significant difference between the conditions.

## Conflict of Interest

The authors declare no conflict of interest.

## Supporting information

Supporting Information

Supplemental Movie 1

Supplemental Movie 2

## Data Availability

The data that support the findings of this study are available from the corresponding author upon reasonable request.
