# Supplementary material for: Acoustofluidic Stimulation of Functional Immune Cells in a Microreactor
Source: Adv Sci (Weinh). 2022 Mar 25;9(16):2105809. doi: 10.1002/advs.202105809 (PMC9165514; doi:10.1002/advs.202105809)
Supplement: Supplementary file 1 — Supporting Information [file ADVS-9-2105809-s003.pdf]

Supporting Information

Acoustofluidic Stimulation of Functional Immune Cells in a Microreactor

Seunggyu Kim[1], Hyeono Nam[1], Beomseok Cha[2], Jinsoo Park[2,*], Hyung Jin Sung[1,*], and Jessie S. Jeon[1,*]

[1]*Department of Mechanical Engineering, Korea Advanced Institute of Science and Technology, Daejeon 34141, Republic of Korea*
[2]*School of Mechanical Engineering, Chonnam National University, Gwangju 61186, Republic of Korea*
*Corresponding authors

Supporting information includes:

Figure S1. Device fabrication process.

Figure S2. Optical image of the fabricated SAW microreactor for dynamic cell culture.

Figure S3. Power reflection spectrums of fabricated IDTs indicating the resonance frequencies as 96.7 MHz.

Figure S4. Temperature elevation analysis of the system under SAW at varying parameters in terms of applied voltages (3.64 and 2.84 Vp-p) and duty cycles (fixed 500 ms-on period and variable off-periods).

Figure S5. Visualization of ASF in the SAW microreactor with different locations and shapes of acoustic cavities.

Figure S6. 2D PIV analysis for ASF velocity estimation.

Figure S7. Computational visualization of time-averaged- streamlines and virtual cell trajectories in the SAW microreactor at varying initial displacements (ξ_0 = 200, 400, and 600 pm).

Figure S8. Measurements for the diameter of NK-92 cells.

Figure S9. Computational calculation of fluid shear stress on the cells in the SAW microreactor.

Figure S10. Investigation of NK-92 cell vitality by (A) Trypan blue exclusion assays for cell viability, (B) EdU incorporation assays for cell proliferation, and (C) F-actin immunofluorescence staining for cytoskeleton.

Figure S11. A schematic of proposed signaling pathway involved in NK cell activation.

Supplementary Table 1. Parameters for the characterization of ASF in the SAW microreactor.

Supplementary Movie 1. Visualization of particle pathlines.

Supplementary Movie 2. Computational visualization of virtual cell trajectories.

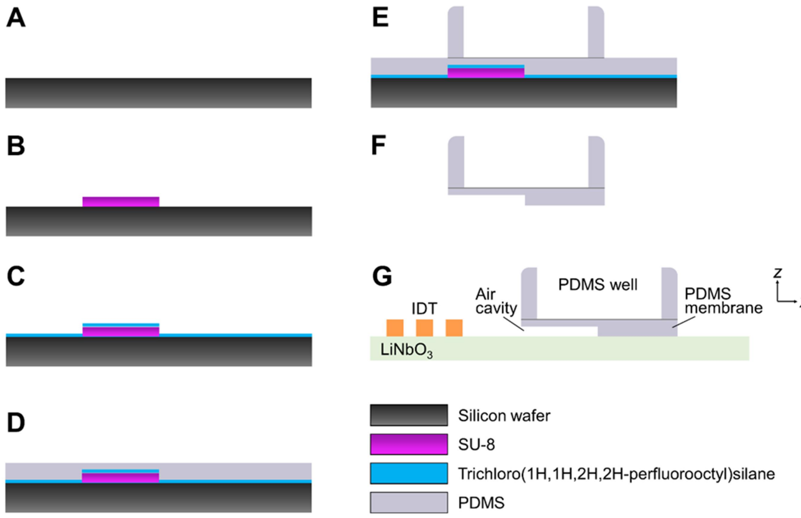

Figure S1. Device fabrication process. (A-D) PDMS membrane having an acoustic cavity was fabricated by depositing a PDMS mixture onto a silane-coated and SU-8 patterned silicon wafer. (E-G) After bonding a perforated PDMS well to the PDMS membrane, the PDMS device was placed onto an IDT-deposited $LiNbO_3$ substrate and the system was utilized in the experiment.

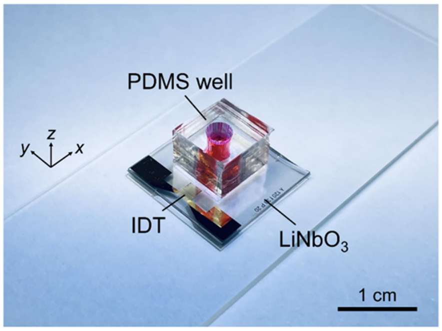

Figure S2. Optical image of the fabricated SAW microreactor for dynamic cell culture. The microreactor consists of a disposable PDMS well and an IDT-deposited $LiNbO_3$ substrate.

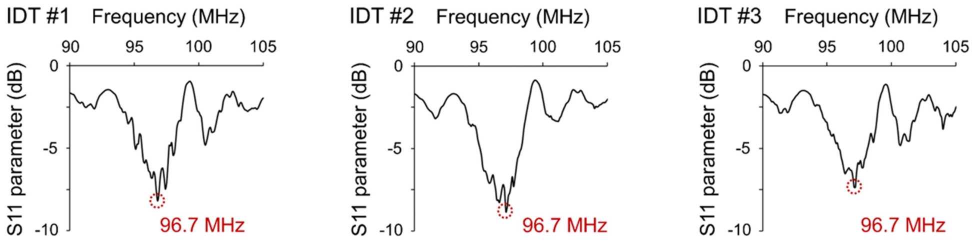

Figure S3. Power reflection spectrums of fabricated IDTs indicating the resonance frequencies as 96.7 MHz.

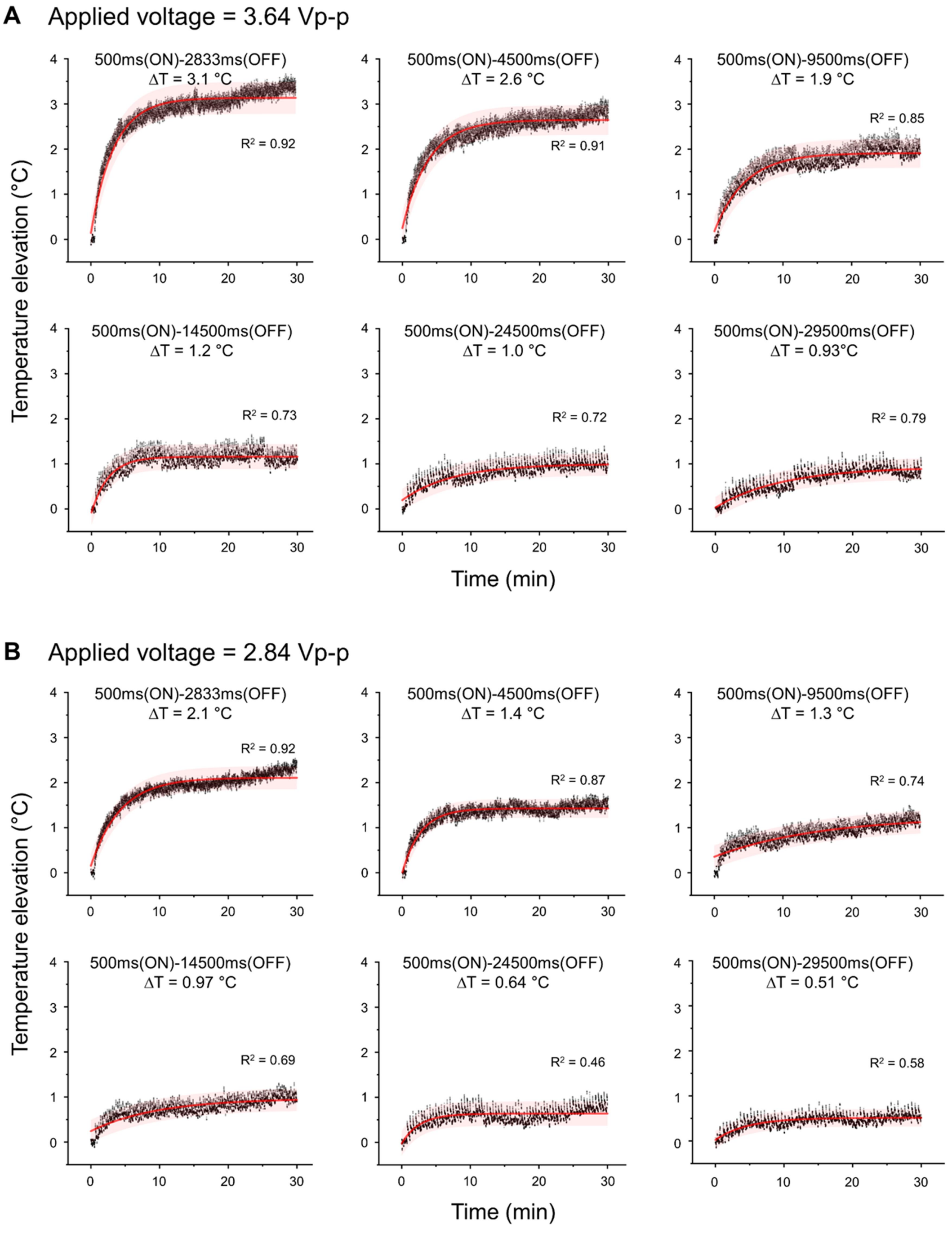

Figure S4. Temperature elevation analysis of the system under SAW at varying parameters in terms of applied voltages (3.64 and 2.84 Vp-p) and duty cycles (fixed 500 ms-on period and variable off-periods). The red line and pink band represent fitted exponential curve and 95% prediction band of measurements, respectively.

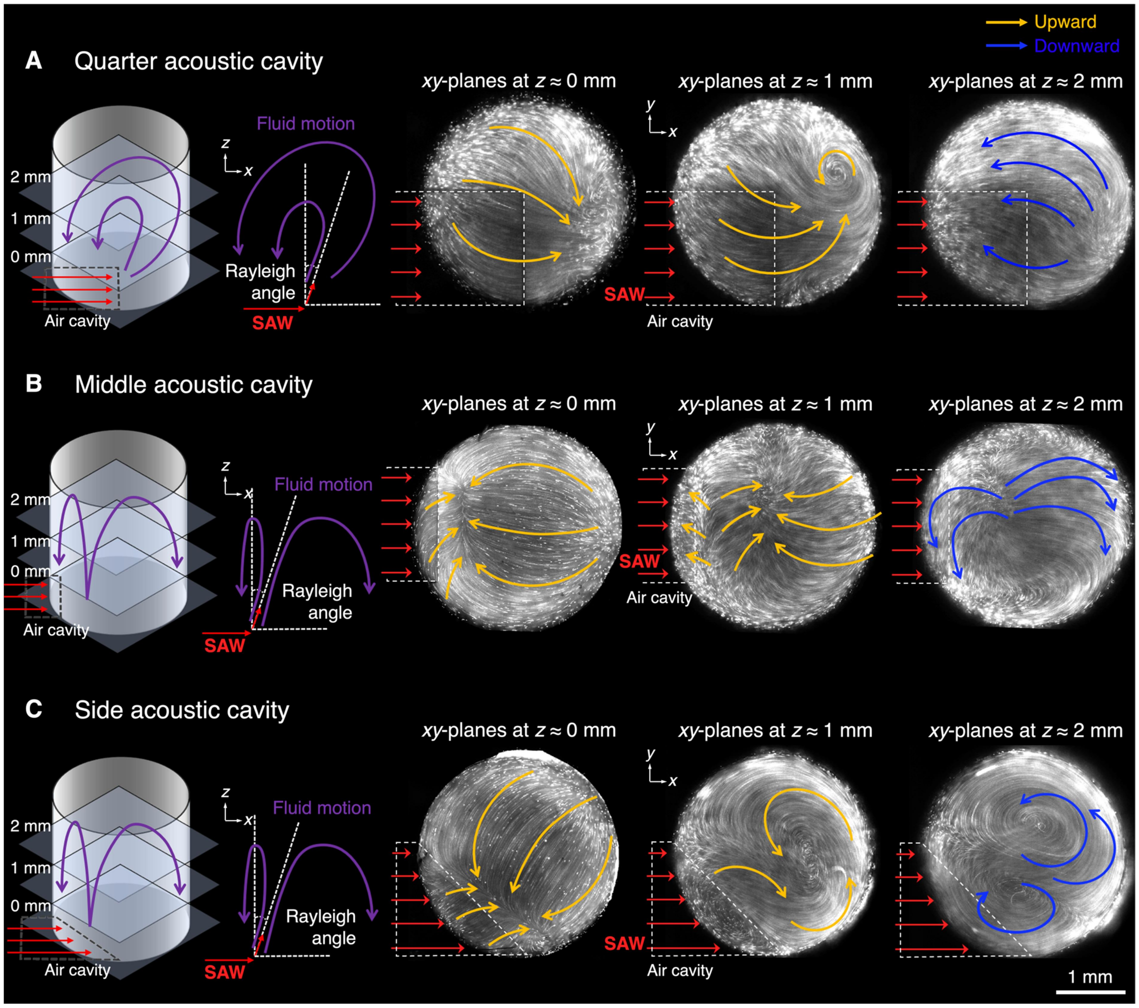

Figure S5. Visualization of ASF in the SAW microreactor with different locations and shapes of acoustic cavities. The one-directional 3D ASF was generated in the microreactor with a quarter acoustic cavity (A) but the two-directional 3D ASF was generated in the microreactors with middle (B) and side (C) acoustic cavities. The shown microscope images are 10-stacked-images in respective *xy*-planes at $z \approx 0$, 1, and 2 mm from the bottom PDMS membrane. The yellow and blue lines in the images represent upward and downward flows, respectively.

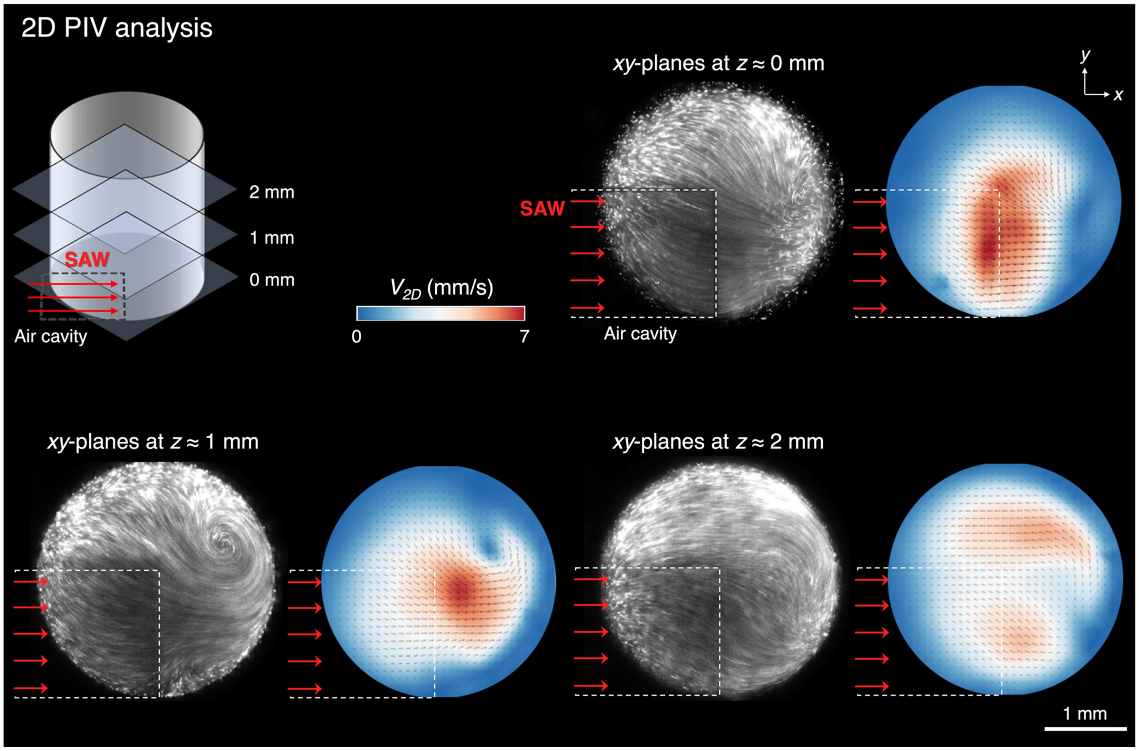

Figure S6. 2D PIV analysis for ASF velocity estimation. The microscope images are 10-stacked-images in respective *xy*-planes at $z \approx 0$, 1, and 2 mm from the bottom PDMS membrane, and corresponding PIV images in the *xy*-planes are shown.

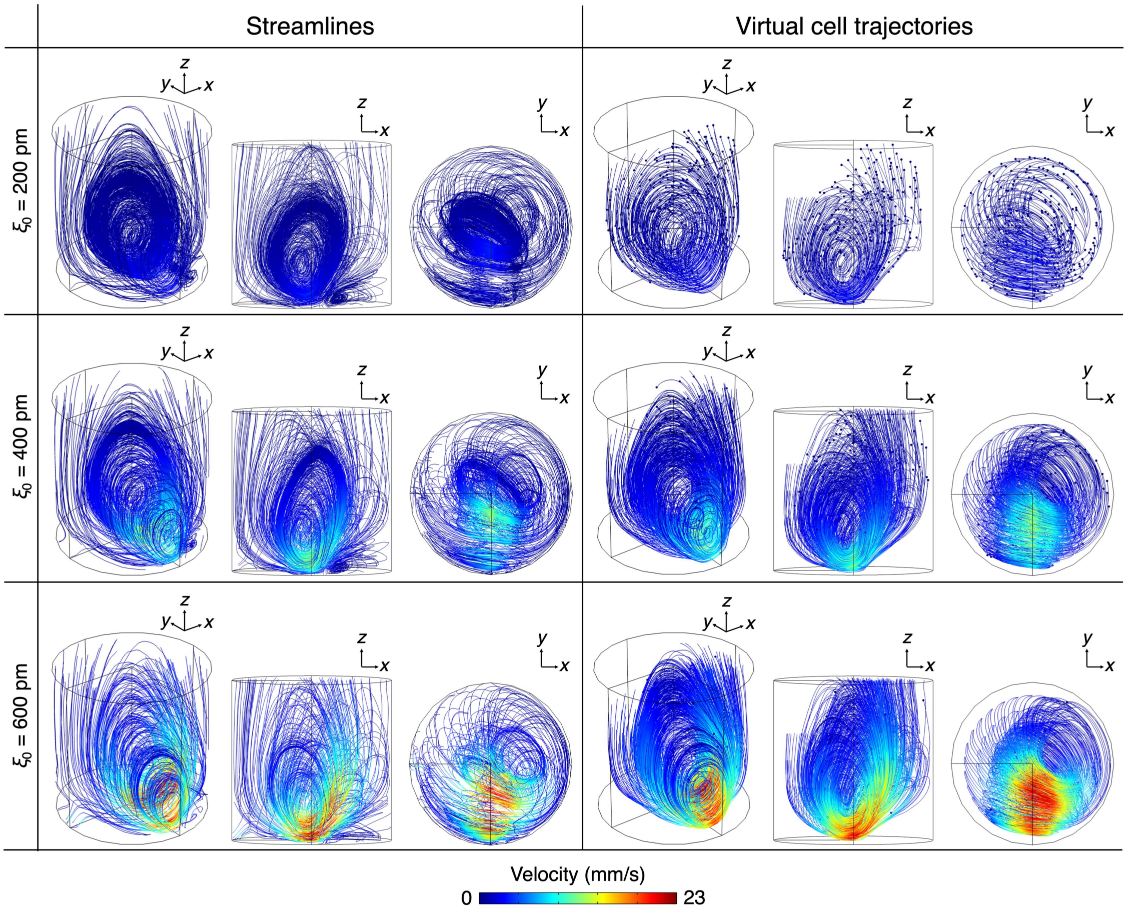

Figure S7. Computational visualization of time-averaged- streamlines and virtual cell trajectories in the SAW microreactor at varying initial displacements (ξ_0 = 200, 400, and 600 pm).

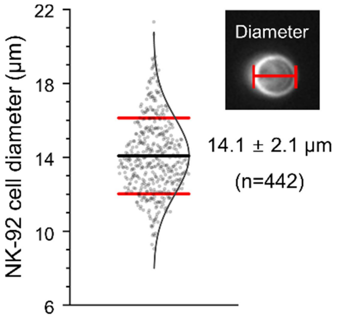

Figure S8. Measurements for the diameter of NK-92 cells. The black line and red lines represent average ± standard deviations (n = 442).

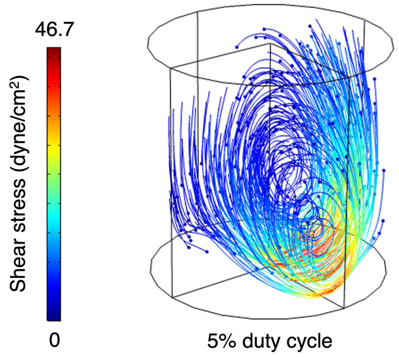

$$\text{Shear stress } (SS) = \frac{3}{2}\frac{\mu V}{r}$$

$$\text{Maximum } SS \approx 46.7 \text{ dyne/cm}^2$$

μ = dynamic viscosity of 37℃ water (≈ 0.691 mPa·s)
r = particle radius (≈ 7.05 μm)
V = computed particle velocity magnitude

Figure S9. Computational calculation of fluid shear stress on the cells in the SAW microreactor. The SAW condition and cells were assumed to be 5% duty cycle at ξ_0 = 600 pm and 14 μm-diameter solid microparticles, respectively.

Supplementary Table 1. Parameters for the characterization of ASF in the SAW microreactor.

Description	Nomenclature	Value	Unit
PIV			
Fluorescent particle density	ρ_p	1,050	kg/m³
Fluorescent particle diameter	d_p	2.00	μm
Dynamic viscosity of water at 20°C	μ_f	1.001	mPa·s
Radius of microwell	R	1.50	mm
Maximum flow velocity at bottom plane obtained from PIV	$V_{2D@z\approx 0}$	≈ 7.00	mm/s
CFD			
Particle density	ρ_c	1,060	kg/m³
Particle radius	r	7.05	μm
Dynamic viscosity of water at 37°C	μ	0.691	mPa·s
Water density at 37°C	ρ_f	993.3	kg/m³
Computed particle velocity magnitude	V	-	mm/s

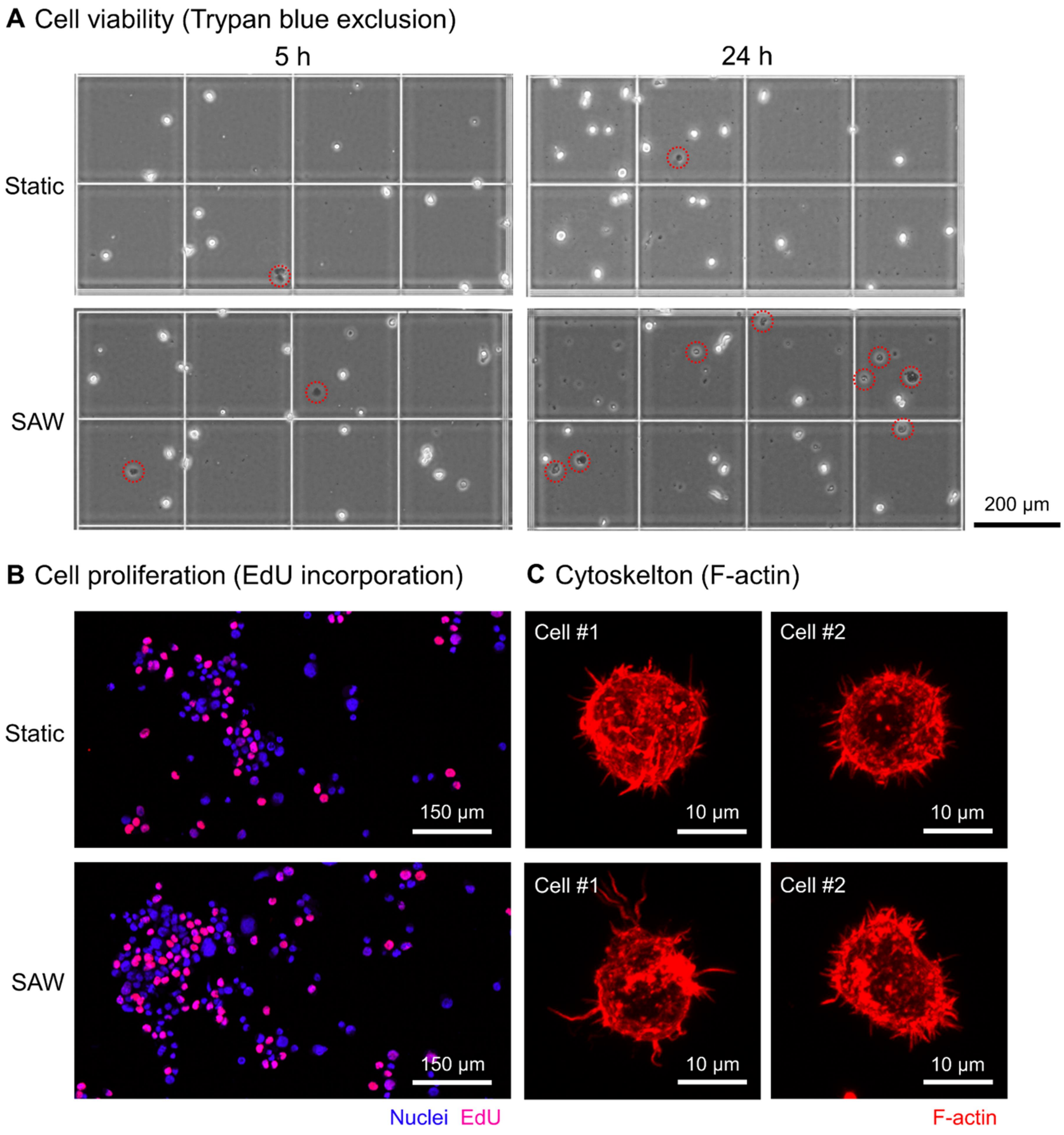

Figure S10. Investigation of NK-92 cell vitality by (A) Trypan blue exclusion assays for cell viability, (B) EdU incorporation assays for cell proliferation, and (C) F-actin immunofluorescence staining for cytoskeleton. Red dotted circles in (A) indicate dead NK-92 cells.

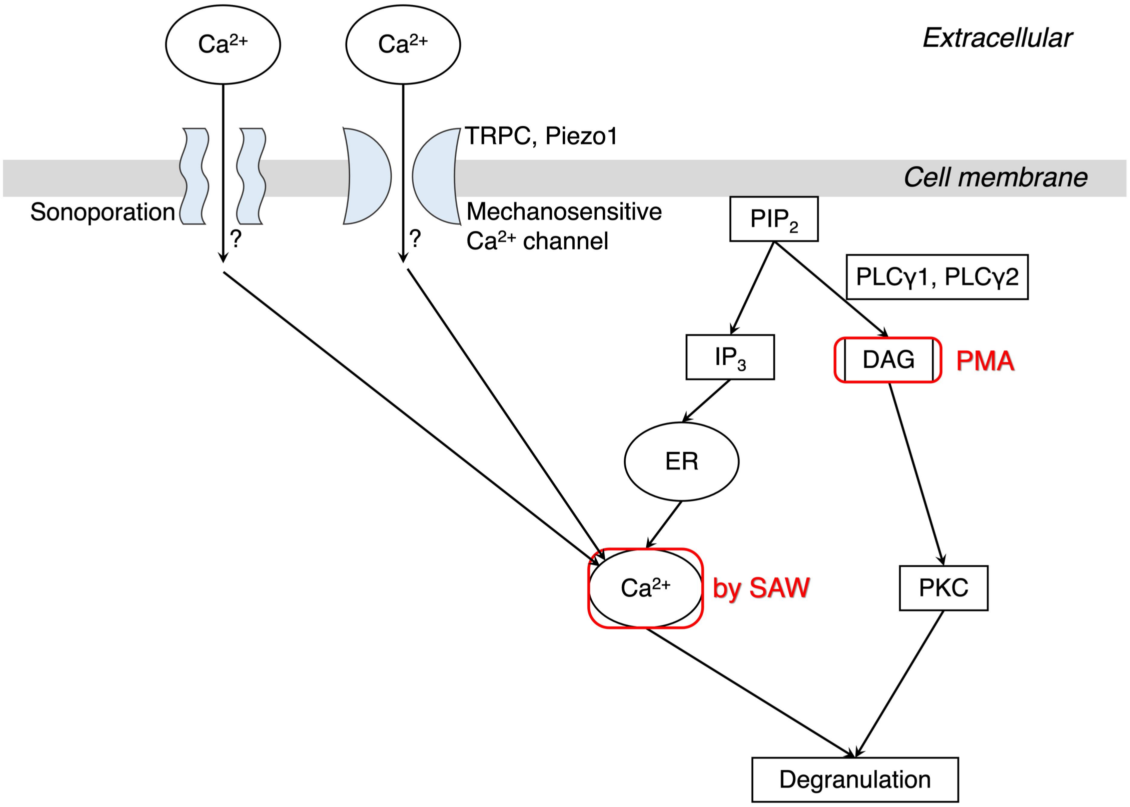

Figure S11. A schematic of proposed signaling pathway involved in NK cell activation. The experimental results suggest that both SAW-derived Ca^{2+} influx and PMA act as inducers involved in the signaling pathways of NK cell activation, which might substitute two indispensable transductions in the pathways such as release of Ca^{2+} from endoplasmic reticulum and activation of protein kinase C, respectively. PMA: Phorbol 12-myristate 13-acetate; DAG: diacylglycerol; PKC: protein kinase C; PLCγ: phospholipase C γ; PIP_2: phosphatidylinositol 4,5-bisphosphate; IP_3: inositol triphosphate; ER: endoplasmic reticulum; TRPC: transient receptor potential cation.

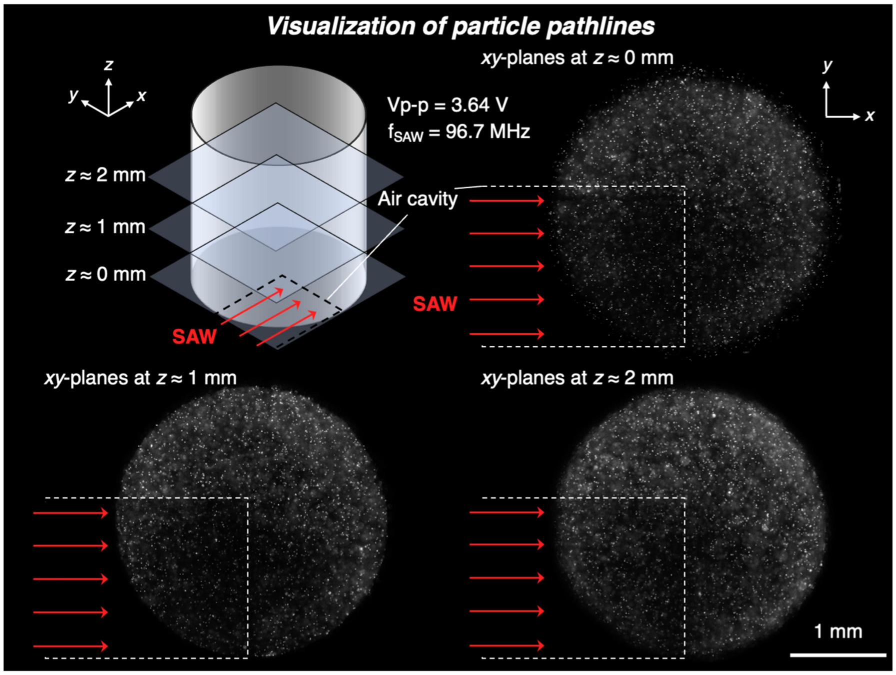

Supplementary Movie 1. Visualization of particle pathlines. The 3D vortical flow is formed in the microreactor under continuous SAW excitation of 96.7 MHz at 3.64 Vp-p, and it is captured with a high-speed camera. The pathlines of 2 μm-diameter particles at three different depths of $z \approx 0$, 1, and 2 mm are presented.

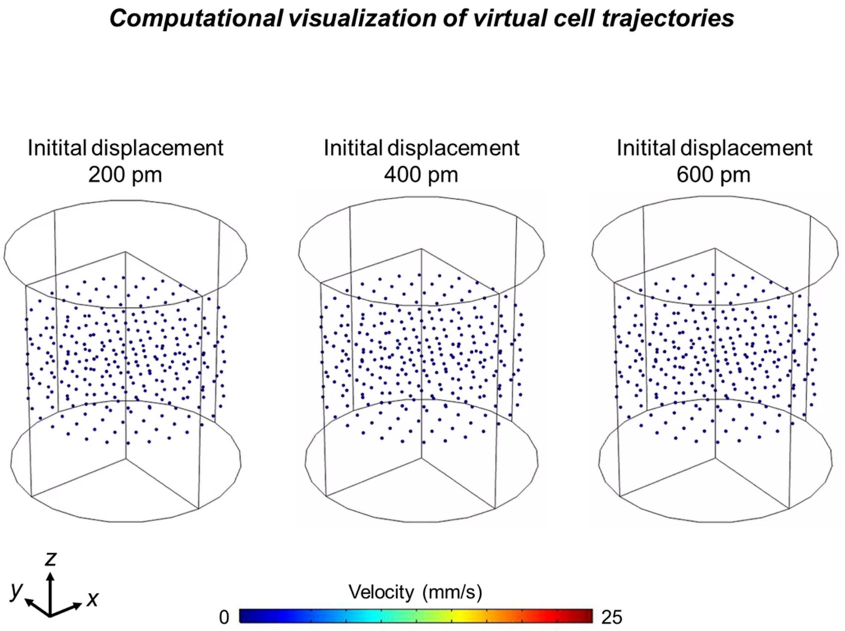

Supplementary Movie 2. Computational visualization of virtual cell trajectories. The 3D virtual particle trajectories formed by the SAW-induced ASF are numerically calculated and visualized at varying initial displacements (ξ_0 = 200, 400, and 600 pm).
